# A rare case of traumatic eye injury

**DOI:** 10.11604/pamj.2022.41.323.34020

**Published:** 2022-04-21

**Authors:** Sharjeel Hafiz Khan, Shyam Vinodrao Chaudhari

**Affiliations:** 1 Department of Forensic Medicine, Narendra Kumar Prasadrao (NKP) Salve Institute of Medical Sciences and Research Centre and Lata Mangeshkar Hospital (LMH), Nagpur, India,; 2 Department of Cardiorespiratory Physiotherapy, Vidya Shikshan Prasarak Mandal´s (VSPM´s) College of Physiotherapy, Nagpur, Maharashtra, India

**Keywords:** Trauma, medicolegal case, clinical forensic medicine unit

## Image in medicine

We report an unusual medico legal case of traumatic right eye injury in the clinical forensic medicine unit of the casualty of a medical college in Central India. The case happened to be a 11-year-old female who accidentally pierced the hooked blunt end of 8 feet iron rod while playing the courtyard. Initially our assessment revealed that there might be profound damage to the eyeball but interestingly the eye structures were intact making this case of an atypical category. Accordingly, the medico legal case was registered in the clinical forensic medicine unit. The patient presented with the complains of pain and swelling in the right eye with an inability to open the eye. The X-ray of the right orbit with the anteroposterior (AP) and lateral view showed the iron rod blunt end being hooked inside the right orbit. On examination the blunt end of the rod was lodged in the fornix between the upper eye lid above with sclera and conjunctiva below in posteriorly upwards direction. The rod was pulled out judiciously by the ophthalmic consultant in the operation theatre. The patient was admitted and the eye was dressed. The patient was discharge on third day on oral antibiotic and painkiller. The visual acuity at the time of discharge was 6/6 in bilateral eye. Interestingly all the vital parts of the eye were not damage in spite of such a degree of trauma making it an unusual case presentation.

**Figure 1:**
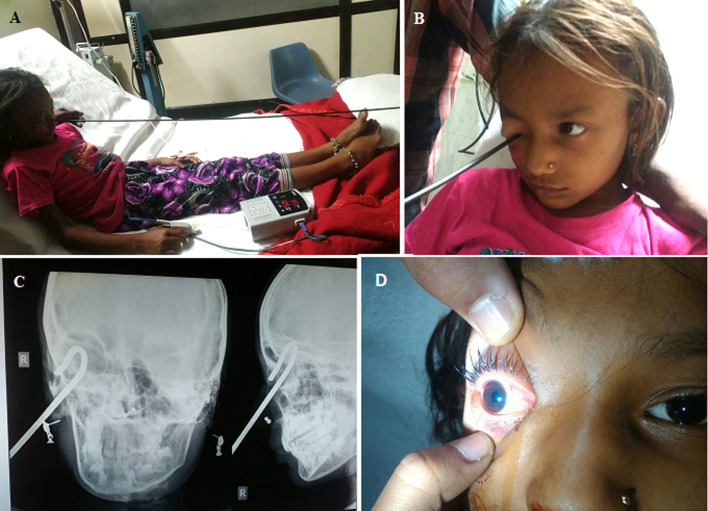
(A,B) traumatic eye injury with iron rod intact; C) X-ray showing iron rod blunt end being hooked inside the right orbit; D) post iron rod removal

